# Human Glomerular Endothelial Cells Treated With Shiga Toxin Type 2 Activate γδ T Lymphocytes

**DOI:** 10.3389/fcimb.2021.765941

**Published:** 2021-11-25

**Authors:** David Antonio Rosso, Micaela Rosato, Fernando Daniel Gómez, Romina Soledad Álvarez, Carolina Maiumi Shiromizu, Irene Angélica Keitelman, Cristina Ibarra, María Marta Amaral, Carolina Cristina Jancic

**Affiliations:** ^1^ Instituto de Medicina Experimental–Consejo Nacional de Investigaciones Científicas y Técnicas (CONICET)–Academia Nacional de Medicina., Buenos Aires, Argentina; ^2^ Laboratorio de Fisiopatogenia, Departamento de Fisiología, Instituto de Fisiología y Biofísica Bernardo Houssay (IFIBIO Houssay–Consejo Nacional de Investigaciones Científicas y Técnicas (CONICET)), Facultad de Medicina, Universidad de Buenos Aires, Buenos Aires, Argentina; ^3^ Departamento de Microbiología, Parasitología e Inmunología, Facultad de Medicina, Universidad de Buenos Aires, Buenos Aires, Argentina

**Keywords:** γδ T cells, hemolytic uremic syndrome, Shiga toxin type 2, inflammation, Th1-like profile

## Abstract

The hemolytic uremic syndrome associated with diarrhea, a consequence of Shiga toxin (Stx)-producing *Escherichia coli* infection, is a common cause of pediatric acute renal failure in Argentina. Stx type 2a (Stx2a) causes direct damage to renal cells and induces local inflammatory responses that involve secretion of inflammatory mediators and the recruitment of innate immune cells. γδ T cells constitute a subset of T lymphocytes, which act as early sensors of cellular stress and infection. They can exert cytotoxicity against infected and transformed cells, and produce cytokines and chemokines. In this study, we investigated the activation of human peripheral γδ T cells in response to the incubation with Stx2a-stimulated human glomerular endothelial cells (HGEC) or their conditioned medium, by analyzing in γδ T lymphocytes, the expression of CD69, CD107a, and perforin, and the production of TNF-α and IFN-γ. In addition, we evaluated by confocal microscopy the contact between γδ T cells and HGEC. This analysis showed an augmentation in cellular interactions in the presence of Stx2a-stimulated HGEC compared to untreated HGEC. Furthermore, we observed an increase in cytokine production and CD107a expression, together with a decrease in intracellular perforin when γδ T cells were incubated with Stx2a-treated HGEC or their conditioned medium. Interestingly, the blocking of TNF-α by Etanercept reversed the changes in the parameters measured in γδ T cells incubated with Stx2a-treated HGEC supernatants. Altogether, our results suggest that soluble factors released by Stx2a-stimulated HGEC modulate the activation of γδ T cells, being TNF-α a key player during this process.

## Introduction

Hemolytic uremic syndrome (HUS) is a late acute onset of symptoms that can appear after an initial intestinal infection with Shiga Toxin (Stx)-producing *Escherichia coli* (STEC). While some STEC strains can cause severe diseases, others are only associated with mild diarrhea or no disease at all (Coombes et al., 2011). This is due in part to the variations in the genetic background of the virulence genes in the different strains. STEC O157:H7 has been considered the strain most associated with HUS outbreaks (Carter et al., 2021), but there are other serotypes such as STEC O145 that are associated with human disease and have also been the cause of reported outbreaks worldwide (Carter et al., 2021). As well, *Escherichia coli* strain belonging to serotype O104:H4 was responsible for the outbreak of HUS and bloody diarrhea that began in northern Germany, which spread rapidly locally (Bielaszewska et al., 2011; Frank et al., 2011) and subsequently in 15 other countries (Rasko et al., 2011). The particular features of this outbreak were that it affected mainly adult women and provoked severe neurological complications and, it has been reported, several fatal cases (Frank et al., 2011). Of mention, the O104:H4 outbreak strain was an enteroaggregative *Escherichia coli* strain rather than a typical enterohemorrhagic one (Rasko et al., 2011). In addition, a series of outbreaks of infection with enterohemorrhagic *Escherichia coli* O157:H7 occurred in Japan in 1996, the largest outbreak occurring in primary schools in Osaka Prefecture, where more than 7,500 cases were reported (Terajima et al., 2014). The condition of Japan infection was not exactly the same as in continental Europe, where non-O157 STEC serotypes were more common than infections with O157:H7 STEC (Blanco et al., 1992; Caprioli and Tozzi, 1998). In addition, between 2000 and 2012, there were 12 other outbreaks that appear to have resulted from consumption of contaminated foods. Interestingly, other than O157:H7 STEC were characterized, such as O111:H8 (Terajima et al., 2014). In accordance with the data described, in Argentina, HUS is highly prevalent, being the most common cause of acute renal failure and the second cause of chronic renal failure in children younger than 5 years old (Repetto, 1997). Furthermore, it is one of the leading causes of renal transplants in childhood, having STEC O157:H7 as the main pathogen involved. Stx type 1 and type 2 (Stx1 and Stx2) produced by STEC O157:H7 and other strains are considered the main and essential virulence factors associated with HUS that trigger kidney damage in patients. Of mention, STEC strains expressing Stx2 are the most common etiologic pathogen responsible for severe cases of HUS in Argentina, and the subtype Stx2a causes more serious illnesses than strains encoding Stx2c (Fitzgerald et al., 2019). Of note, Stx2 is classified into several subtypes (i.e., Stx2a, Stx2b, Stx2c, Stx2d, Stx2e, Stx2f, and Stx2g) (Scheutz et al., 2012). STEC with Stx2a was more frequently isolated from HUS patients than the strains with the other Stx2 subtypes (Friedrich et al., 2002; Persson et al., 2007). Regarding the toxin structure, the Stxs contain two subunits, A and B, organized as a pentameric ring of identical B-subunits non-covalently associated with a single A-subunit (Lee et al., 2016). The B-subunits allow the interaction with Stx receptor, and the A-subunit has the enzymatic activity involved in the toxic effect. Once the toxin interacts with its receptor, the membrane glycolipid, globotriaosylceramide (Gb3, also known as CD77), the toxin is internalized and suffers a retrograde intracellular trafficking. By the interaction with the pentameric B-subunits, Gb3 cross-linking, it is thought to trigger receptor-mediated endocytosis (Sandvig et al., 2014). Following the internalization, the toxin is sequentially delivered from an early endosome to the trans-Golgi network, then to the Golgi apparatus, and finally to the endoplasmic reticulum (Sandvig et al., 1992). During the transport to the endoplasmic reticulum, Stx A-subunits dissociate from the B-subunits through a mechanism that involves proteolysis and disulfide bond reduction (Garred et al., 1995). Once dissociated, fragments of the A-subunits associate with chaperones present in the endoplasmic reticulum and then retrotranslocate to the cytosol (LaPointe et al., 2005). Once in the cytoplasm, toxin A-fragments appear to re-fold into their active conformation (Hazes and Read, 1997). All this process results in host cell protein synthesis inhibition, activation of the ribotoxic and endoplasmic reticulum stress responses, and in the induction of apoptosis (of epithelial, endothelial, lymphoid and myeloid cells), autophagy and increased expression of pro-inflammatory cytokines and chemokines (Johannes and Römer, 2010), which contribute to tissue damage in the colon and the development of HUS and central nervous system complications. HUS is clinically characterized by microangiopathic hemolytic anemia, thrombocytopenia, and variable degrees of kidney injury (Alconcher et al., 2018). During the hemorrhagic colitis stage, disruption of the mucosa allows the Stx to enter the bloodstream and to target tissues expressing its receptor, which is present in the microvasculature of several organs, mainly the kidney and central nervous system (Boyd and Lingwood, 1989; Fujii et al., 2008). Of note, the kidney is the most affected organ by Stx2a due to its high expression of Gb3 receptor and its physiological function of filtering large volumes of blood in which the toxin could be present, increasing the probability of contact between Stx2a and Gb3 (Obrig, 2010). Once in contact with the glomerular endothelial cells, Stx2a activates them and causes a switch into a proinflammatory state by increasing the expression of adhesion molecules and the secretion of cytokines and chemokines, thus promoting the recruitment of leukocytes (Ramos et al., 2007). Interestingly, macropinocytosis increases toxin endocytosis by intestinal epithelial cells and also stimulates toxin transcellular transcytosis. Thus, macropinocytosis might be responsible for toxin uptake by Gb3-free intestinal epithelial cells and transcytosis (Malyukova et al., 2009). Once within the bloodstream, most of the toxin circulate associated to blood cells such as leukocytes (Te Loo et al., 2011), platelets, and aggregates between these cells (Ståhl et al., 2009), and red blood cells (Arvidsson et al., 2015). Also, it has been reported that the binding of toxin to blood cells activates them and induces the shedding of microvesicles, which are pro-inflammatory, pro-thrombotic (Arvidsson et al., 2015), and, importantly, transport the toxin to its target organ. This has been suggested to be one of the main mechanisms of toxin-induced systemic and targeted organ injury (Karpman et al., 2017). Regarding the innate cells participating in the pathogenesis of HUS, it has been reported that monocytes, NK cells, and neutrophils are involved (Ramos et al., 2007; Ramos et al., 2016). Nevertheless, there is no evidence in humans about the role of γδ T cells, which are lymphocytes of the innate and adaptive immune response. γδ T cells represent 1–10% of the total T cell population in peripheral blood (Fonseca et al., 2020), and those expressing Vγ9Vδ2 TCR constitute the main circulating γδ T cell subset in healthy humans (Silva-Santos et al., 2015). As other innate immune cells, γδ T lymphocytes can infiltrate tissues (Fay et al., 2016), and in kidney injury, γδ T cells can be potentially recruited as they express the fractalkine receptor CX3CR1 (Nishimura et al., 2002), which was demonstrated to be involved in the recruitment of NK cells and monocytes to this organ (Malyukova et al., 2009). Interestingly, Vγ9Vδ2 T cells do not recognize antigens presented in the context of the major complex histocompatibility molecules. Instead, these cells display a unique response to non-peptide prenyl-pyrophosphate antigens, called phosphoantigens, such as isopentenyl pyrophosphate (IPP), which is produced by eukaryotic cells, and they can also recognize those that are produced by prokaryotic cells such as (E)-4-hydroxy-3-methyl-but-enyl pyrophosphate (HMBPP) (Poupot and Fournié, 2004). IPP is overexpressed by cells suffering malignant transformation, allowing them to be targeted by Vγ9Vδ2 T cells, after the interaction with the molecule BTN3A1 (Harly et al., 2015). Apart from the phosphoantigens, γδ T cells can be activated by cytokines, molecules induced by stress, and microbes’ components, among other molecules (Shiromizu and Jancic, 2018). Once activated, γδ T cells release cytokines, such as IFN-γ and TNF-α, and granules containing granzymes and perforins that lead to the cytotoxicity of target cells. Additionally, they can exert the non-secretory mechanism of cytotoxicity by the interaction between Fas and FasL.

In this work, we aimed to investigate the γδ T cell response to either the presence of human glomerular endothelial cells (HGEC) stimulated with highly purified Stx2a, or its conditioned medium, to elucidate a possible role of γδ T cells during the pathogenesis of HUS.

## Methods

The experimental protocols performed were reviewed and approved by the Biosafety and Research Review boards of the *Instituto de Medicina Experimental–CONICET, Academia Nacional de Medicina* and the Ethical Committee of the *Institutos de la Academia Nacional de Medicina* and the *Universidad de Buenos Aires*. The methods were carried out following the approved guidelines. The participants provided their written informed consent to participate in this study.

### Peripheral Blood γδ T Lymphocyte Isolation

γδ T cells were isolated from heparinized human blood from healthy donors, who gave written informed consent, by centrifugation on Ficoll-Hypaque and positive selection using magnetic microspheres covered with anti-TCR γδ antibodies, according to the manufacturer’s instructions (Miltenyi Biotec, Germany). After purification, cells were resuspended in RPMI 1640 supplemented with 10% heat-inactivated fetal bovine serum (FBS), penicillin (100 U/ml), and streptomycin (100 µg/ml). Cells were analyzed by flow cytometry (FACSCalibur, Beckton Dickinson, San Jose, CA, USA) to guarantee that γδ T cell purity was >98% and monocyte contamination <2% (**Supplementary Material, Figure 1**). The purification procedure did not affect cell activation; this was evaluated by analyzing the expression of CD69 and the production of TNF-α in the presence or absence of the agonist HMBPP (**Supplementary Material, Figure 2**). The details about donors are listed in **Supplementary Material, Table 1A**.

### Human Glomerular Endothelial Cell Culture

HGEC were isolated from healthy areas of kidney fragments from patients undergoing nephrectomies as a consequence of segmental uropathies or tumors in one pole and normal creatinine. The procedure was performed at Hospital Nacional Alejandro Posadas, Buenos Aires, Argentina. Endothelial cells were isolated as was previously described (Amaral et al., 2013). Once obtained, cells were grown in M199 media supplemented with 20% FBS, 3.2 mM L-glutamine, 100 U/ml penicillin/streptomycin, and 25 µg/ml endothelial cell growth supplement. For growth-arrested conditions, a medium with 10% of FBS and without endothelial cell growth supplement was employed. For the experiments, cells were used between 2 and 7 passages, after characterization for von Willebrand factor and platelet/endothelial cell adhesion molecule 1 (PECAM-1) positive expression (Amaral et al., 2013). The details about donors are listed in **Supplementary Material, Table 1B**.

To treat HGEC, highly purified Stx2a (provided by Phoenix Laboratory, Tufts Medical Center, Boston, MA, USA) was used. Lipopolysaccharide content on Stx2a was checked by Limulus amebocyte lysate test (<10 pg/ml).

### Reagents and Antibodies

The reagents employed in this work are listed in **Supplementary Material, Table 2**.

The description of the different methodologies employed in this work is in **Supplementary Material**.

## Results

### Stx2a-Stimulated Human Glomerular Endothelial Cells Promote the Activation of γδ T Lymphocytes

**Figure 1:**
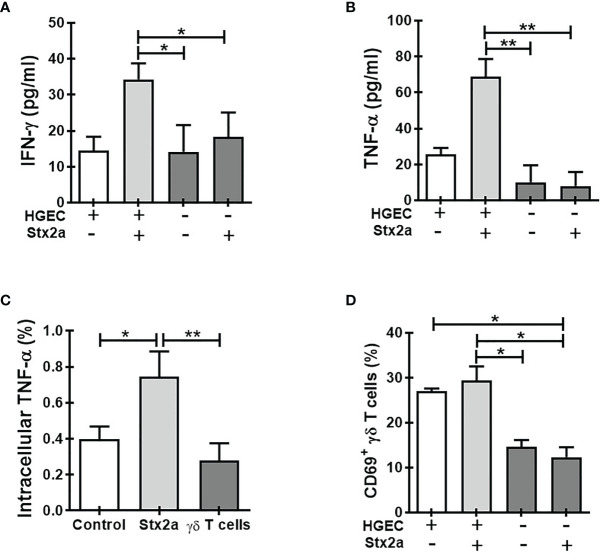
Co-culture of γδ T cells with Stx2a-stimulated HGEC induce its activation. γδ T cells were cultured overnight at 37°C in the absence or presence of HGEC, treated or not previously with Stx2a (0.01 ng/ml, 24 h). γδ T cells were resuspended in M199 supplemented with 10% FBS. After culture, supernatants and cells were collected. **(A)** IFN-γ and **(B)** TNF-α production in supernatants was measured by ELISA, n=7 and n=5, respectively. Biological replicates. Kruskal-Wallis test, with Dunn’s multiple comparisons test. **(C)** Intracellular cell staining of TNF-α in γδ T lymphocytes. Control (open bar) represents γδ T cell culture with HGEC non-treated with Stx2a, n=8. Biological replicates. Kruskal-Wallis test, with Dunn’s multiple comparisons test. **(D)** CD69 expression in γδ T cells, was analyzed by flow cytometry, n=5. Biological replicates. Kruskal-Wallis test, with Dunn’s multiple comparisons test. Results are shown as the mean ± SEM, *p < 0.05, **p < 0.01.

In this study, we focused on the role of circulating γδ T cells in the pathogenesis of the HUS. We speculated that γδ T cells could detect the damages generated by Stx2a on endothelial cells once accesses to the blood circulation and impacts on kidneys. We propose that γδ T lymphocytes sense cell damage, and then they activate and put in place different mechanisms to exacerbate local inflammation. Therefore, we evaluated the effect of HGEC stimulated or not by Stx2a on the functionality of γδ T cells. For this purpose, we incubated human purified γδ T lymphocytes obtained from peripheral blood with confluent cultures of HGEC, previously treated or not with Stx2a (0.01 ng/ml, overnight). As it is shown in **Figure 1**, the presence of Stx2a-treated HGEC increased the secretion of IFN-γ (**Figure 1A**) and TNF-α (**Figure 1B**) by γδ T cells, supporting their differentiation towards a Th1-like profile. Because HGEC are able to produce TNF-α (see **Figure 3B**), we decided to analyze the production of this cytokine in γδ T cells by intracellular cell staining, in addition to the ELISA quantification. The result obtained confirmed that γδ T cells effectively produce TNF-α (**Figure 1C**). Of note, under our experimental conditions, we did not detect a modulation by Stx2a of the level of the activation marker CD69 on γδ T cells (**Figure 1D**), suggesting that γδ T lymphocytes’ responses differ when cultured with Stx2a-stimulated HGEC, compared to IFN-γ and TNF-α secretion.

**Figure 2:**
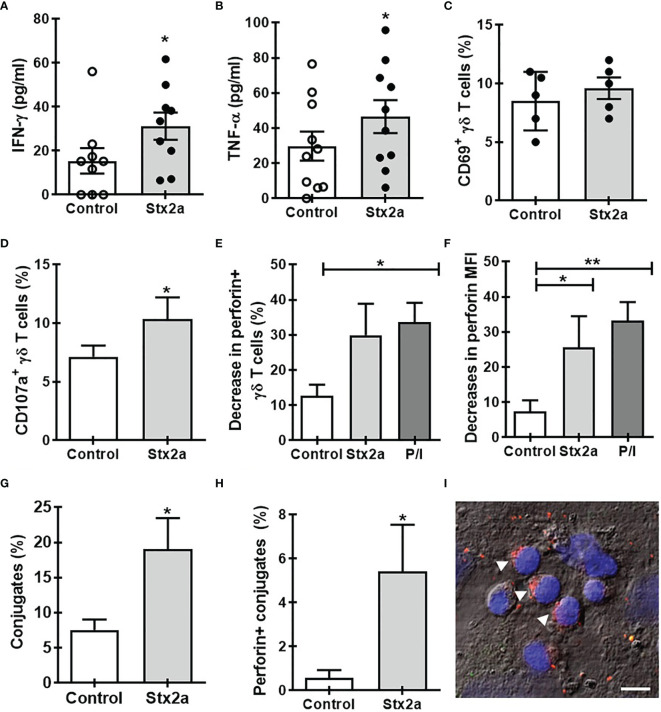
Soluble factors released by Stx2a-stimulated HGEC activate γδ T cells. γδ T cells were cultured overnight at 37°C with conditioned media obtained from confluent monolayers of HGEC, treated (Stx2a) or not (Control) with Stx2a (0.01 ng/ml, 24 h). After overnight incubation, supernatants and cells were collected. **(A, B)** IFN-γ and TNF-α released to the medium determined by ELISA, n=9 and n=10, respectively. Biological replicates. Kruskal-Wallis test, with Dunn’s multiple comparisons test. **(C–F)** Flow cytometry analysis of the expression of CD69 **(C)** (n=5), CD107a **(D)** (n=9), and perforin **(E)** (n=5) and **(F)** (n=10) in γδ T cells. PMA and ionomycin (P/I) were used as a positive control. **(G, H)** γδ T cells were seeded on glass slides previously coated with HGEC treated or not (Control) with Stx2a (0.01 ng/ml, 24 h) (for details see the section *Methods*). After incubation, the number of γδ T cells interacting with HGEC (conjugates) and the expression of perforin were evaluated by confocal microscopy. **(G)** Percentage of cell conjugates in non-treated (Control) or Stx2a-stimulates HGEC (Stx2a) (n=10, technical replicates). **(H)** Percentage of perforin+ γδ T cells in interaction with HGEC non-treated (Control) or treated with Stx2a (Stx2) (n=10, technical replicates). **(I)** Representative image of perforin expression (red) in γδ T cells in co-culture with Stx2a-treated HGEC. For nuclear visualization, cells were stained with ToPro-3 (blue). The figure shows one of two independent experiments performed. Bar: 10 µm. Arrows indicate γδ T cells in which perforin are polarized. Results are shown as the mean ± SEM. *p < 0.05 and **p < 0.01; **(A–D, G, H)** Wilcoxon or Mann-Whitney test. **(E, F)** Kruskal-Wallis with the Dunn’s multiple comparisons posttest.

Then, we speculated that soluble factors released by endothelial cells in the presence or absence of Stx2a could affect the activation of γδ T lymphocytes. To evaluate this hypothesis, we incubated γδ T cells with conditioned media obtained from confluent cultures of HGEC treated or not with Stx2a. As **Figure 2** shows, those supernatants induced the production of IFN-γ (**Figure 2A**) and TNF-α (**Figure 2B**) but did not change the expression of CD69 (**Figure 2C**), similar to that observed in co-culture conditions. This effect was dependent on the activity of Stx2a, since when it was inactivated by heating, the γδ T cells did not change the production of IFN-γ and TNF-α (**Supplementary Figures 3A, B**). Additionally, the conditioned media also prompt an increase in the degranulation of γδ T cells, measured by the expression of CD107a (**Figure 2D**) and intracellular perforin (**Figures 2E, F**). Interestingly, we observed a decrease in the percentage of perforin+ γδ T lymphocytes (**Figure 2E**) and in the median intensity fluorescence for perforin (**Figure 2F**) in the presence of medium coming from Stx2a-stimulated HGEC. To complement the cell degranulation study, we performed an analysis by confocal microscopy by co-culturing γδ T cells with HGEC pretreated or not with Stx2a for 24 h. After co-cultured, cells were intracellularly stained for perforin as described in *Methods* (**Figures 2G–I**). In the samples, we evaluated the capacity of γδ T cells to interact with HGEC pretreated or not with Stx2a, and we analyzed the distribution of perforin in γδ T lymphocytes that were in contact with endothelial cells. As we can observe in **Figure 2G**, there was a high percentage of cell conjugates when HGECs were exposed to Stx2a. Interestingly, γδ T lymphocytes attached to Stx2a-treated endothelial cells display higher levels of perforin compared with those in contact with non-treated HGEC (**Figure 2H**). The image of (**Figure 2I**) shows γδ T lymphocytes with perforin polarized to one side of the cytosol, which is a consequence of the cytoskeleton rearrangement after activation and is the step that takes place before the secretion of perforin towards the target cell in the immunological synapse formed (Trapani and Smyth, 2002; Law et al., 2010).

### TNF-α Plays a Critical Role in the Activation of γδ T Lymphocytes by Conditioned Medium From Stx2a-Stimulated Human Glomerular Endothelial Cells

**Figure 3:**
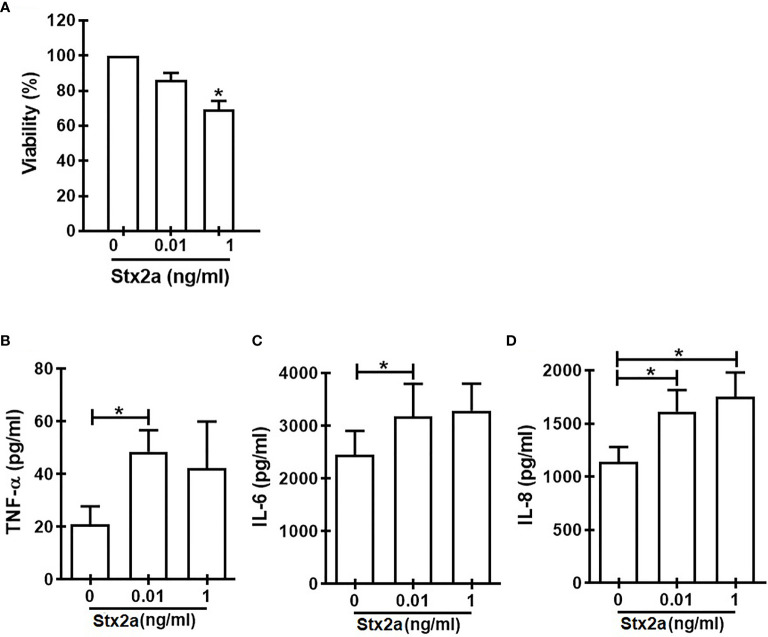
Cytokine secretion by HGEC in response to different concentrations of Stx2a. Confluent monolayers of HGEC were stimulated with different concentrations of Stx2a (0.01 and 1 ng/ml) for 24 h. After incubation, cell supernatants were recovered. **(A)** Percentage of cell viability analyzed by neutral red uptake (n=3, biological replicates). Friedman with the Dunn’s multiple comparisons posttest. **(B)** TNF-α, **(C)** IL-6, and IL-8 **(D)** production measured by ELISA in cell supernatants, n=6 (biological replicates). Friedman with the Dunn’s multiple comparisons posttest. Results are shown as the mean ± SEM. *p < 0.05.

**Figure 4:**
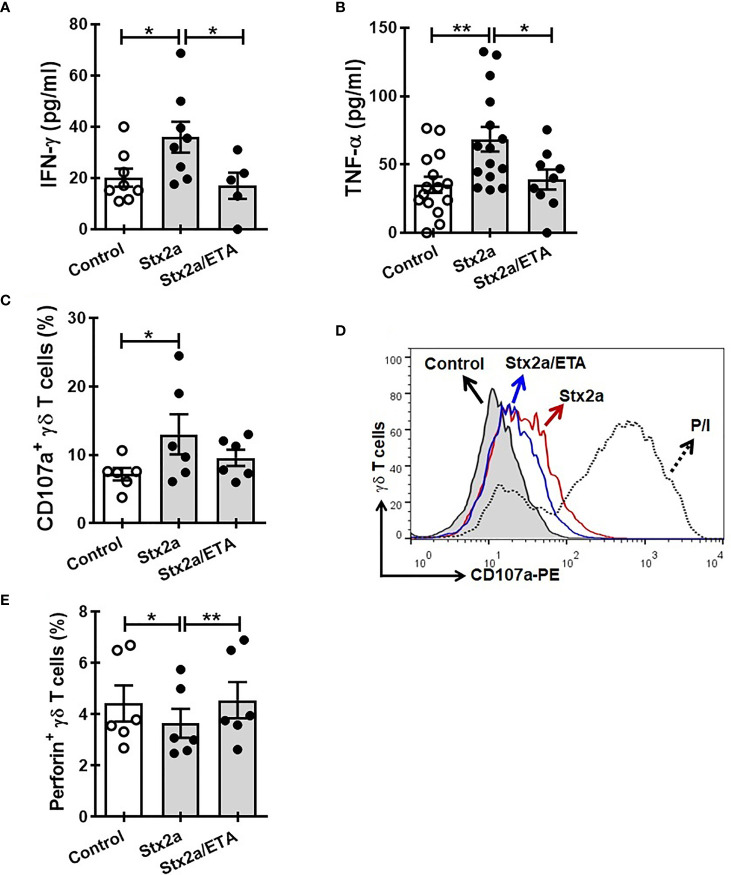
Etanercept impedes the activation of γδ T cells induced by Stx2a-treated HGEC. γδ T cells were cultured overnight at 37°C with a conditioned media obtained from confluent monolayers of HGEC, treated (Stx2) or not (Control) with Stx2a (0.01 ng/ml, 24 h). One group of cells were pre-incubated with Etanercept (ETA: 25 µg/ml, 30 min at 37°C). After overnight incubation, supernatants and cells were collected. **(A, B)** IFN-γ (n=5) and TNF-α (n=9) released to the medium determined by ELISA, respectively. Biological replicates. Kruskal-Wallis test, with Dunn’s multiple comparisons test. **(C, D)** Expression of CD107a in γδ T cells, analyzed by flow cytometry. **(C)** Percentage of CD107+ γδ T cells (n=6). Biological replicates. Kruskal-Wallis test, with Dunn’s multiple comparisons test. **(D)** Representative histogram of one experiment out of six performed. Gray histogram: γδ T cells cultured with conditioned medium from Stx2a-non-treated HGEC (Control); histogram red: γδ T cells cultured with conditioned medium from Stx2a-treated HGEC (Stx2a); histogram blue: γδ T cells cultured in presence of Etanercept (ETA) and with conditioned medium from Stx2a-treated HGEC (Stx2a/ETA); dot line histogram: γδ T cells incubated with PMA and ionomycin (P/I) (positive control). **(E)** Percentage of perforin+ γδ T cells, analyzed by intracellular staining and flow cytometry (n=6). Biological replicates. Friedman test, witn Dunn’s multiple comparison test. Results are shown as the mean ± SEM. *p < 0.05 and **p < 0.01.

In search for a soluble component responsible for the activation of γδ T cells towards a Th1-like profile, we measured some cytokines secreted by HGEC stimulated with different concentrations of Stx2a (0.01 and 1 ng/ml, 24 h). Because we wanted to evaluate the cross-communication between γδ T cells and HGEC, it is noteworthy that it was a requirement for our work to use a concentration of Stx2a that did not significantly affect the HGEC viability but was sufficient to activate them and promote the secretion of cytokines (Álvarez et al., 2019). For that reason, we chose the concentration of 0.01 ng/ml of Stx2a that induced low and not significant cytotoxicity (86% ± 4) (**Figure 3A**) but stimulated cytokine production. **Figure 3** shows that under Stx2a stimulation, HGEC produced TNF-α (**Figure 3B**), IL-6 (**Figure 3C**), and IL-8 (**Figure 3D**). Among those cytokines, it is well-known that TNF-α has the capacity to activate γδ T cells in autocrine or paracrine ways, and even if their concentration is low, it can act on γδ T cells and modulate their activation (Lahn et al., 1998; Rincon-Orozco et al., 2005; Sabbione et al., 2014). Based on this, we decided to assess a blocking experiment in which by using Etanercept we neutralized TNF-α, and then we evaluated the different activation parameters on γδ T lymphocytes, mentioned before. In **Figure 4**, we can observe that the treatment of cells with Etanercept reversed completely the increase in the IFN-γ (**Figure 4A**) and TNF-α (**Figure 4B**) production; and in the CD107a (**Figures 4C, D**) and perforin (**Figure 4E**) expression. These results demonstrated the role of this cytokine in the activation of several mechanisms of γδ T cells that could be implicated in the pro-inflammatory responses induced by Stx2a-stimulated HGEC.

## Discussion

In humans, there are not previous publications reporting the role of γδ T cells during the pathogenesis of HUS, since most of them have studied their role in cattle, calves (Menge et al., 2004), and mice (Obata et al., 2015). In this work, we demonstrated for the first time that Stx2a-stimulated HGEC could modulate the differentiation of γδ T lymphocytes towards a Th1-like profile characterized by the production of IFN-γ and TNF-α. In addition to this phenomenon, there was an increment in the degranulation of γδ T cells, evidenced by the increase in the expression of CD107a (Alter et al., 2004) and the decrease of intracellular perforin. As well, we demonstrated that the interaction of γδ T cells with Stx2a-stimulated HGEC induced the polarization of perforin, which is a key step to allow their posterior secretion together with granzymes towards a target cell (Trapani and Smyth, 2002; Law et al., 2010). This event might contribute to endothelial damage (we are currently studying this hypothesis). Notably, here we demonstrated that the blockage of TNF-α prevents completely the activation of γδ T cells in response to conditioned medium obtained from Stx2a-treated HGEC. Nevertheless, we cannot dismiss that soluble compounds could act in conjunction with cell-to-cell contact, or have a role in favoring intercellular communication, but we need to perform more experiments to elucidate this hypothesis. However, our results clearly show that γδ T cells respond effectively and efficiently to soluble factors released by HGEC stimulated by low concentrations of Stx2a, mainly TNF-α.

Regarding the role of the other cytokines secreted by HGEC studied in this work, such as IL-6 or IL-8, there are few reports describing their functions on human γδ T cells. Based on the limited data published, it seems that some populations of γδ T cells do not react to IL-8 since it has been reported that peripheral blood γδ T cells respond in dose-dependent transendothelial chemotaxis to the CC chemokines such as RANTES, but not to the CXC chemokines as IL-8 (Roth et al., 1998). Other authors have demonstrated that intraepithelial lymphocytes are able to respond to IL-8, although the presence of other chemokines desensitized these cells to IL-8 (Roberts et al., 1997). Based on our results and on the published data mentioned above, we can speculate that in our experimental model, this molecule has no impact on peripheral blood γδ T cell responses. On the other hand, it has been reported that TNF-α and IL-6 upregulate the expression of the chemokine receptors CCR5 and CXCR3 on Vδ2 T cells in patients suffering from rheumatoid arthritis. Interestingly, this increase was abrogated by the administration of neutralizing antibodies against TNF-α (Mo et al., 2017), assigning a leading role to this cytokine in this process, in accordance with our experimental design. In addition and supporting the role of TNF-α during γδ T cell activation, in a model of bacterial infection done by other authors, it was observed the same behavior as we found, and this may be due to the fact that γδ T cells are very sensitive to the effect of TNF-α (Lahn et al., 1998).

Of mention, and in addition to the study of soluble factors as mediators of γδ T cell activation, it is our aim to evaluate the molecules involved in the cell-to-cell interaction between HGEC and γδ T lymphocytes to go further in the study of the participation of these T cells during the initiation and progression of HUS (ongoing project). Additionally, TNF-α released by γδ T cells could also contribute to tissue sensibility to the toxin as this cytokine upregulates Gb3 expression in HGEC (Stricklett et al., 2002). Interestingly, it has been reported that the chemokine fractalkine (FKN; CX3CL1) is involved in many diseases with renal injury (Cockwell et al., 2002; Durkan et al., 2007) including HUS (Ramos et al., 2007). Remarkably, it has been demonstrated that a fraction of monocytes and NK cells, which express the fractalkine receptor CX3CR1+, are diminished in peripheral blood of HUS patients when compared to healthy donors. Moreover, immunohistochemistry on renal biopsies of children with HUS revealed the presence of CX3CR1+ monocytes infiltrating kidneys (Ramos et al., 2007), contributing to the pathogenesis of HUS promoting inflammation events in the glomeruli endothelium, and increasing the initial damage done by Stx. In this sense, during the Stx-induced damage in the glomeruli, the release of pro-inflammatory cytokines promotes leukocyte recruitment, platelet aggregation, and fibrin deposition. Based on this evidence, we can speculate that in γδ T cells, CX3CR1 could play a role by recruiting them to the glomeruli initially affected by the Stx2a and then to promote the γδ T cell participation in the inflammation process, thus contributing to the exacerbation of the endothelial damage and in the pathogenesis of HUS. Altogether, these events could lead to partial or complete vessel occlusion by microthrombi and the consequent microangiopathic hemolytic anemia (Keir et al., 2012). However, more studies are needed to confirm this hypothesis.

Based on our data presented here, and as a first contribution to the knowledge of the role of human γδ T cells in HUS pathogenesis, our results indicate that γδ T cells could be involved in the exacerbation of the renal tissue damage due to the secretion of pro-inflammatory cytokines and to their degranulation. We speculate that once γδ T cells come into contact with stressed endothelial cells, they might recognize signals that allow their activation, leading to start-up of its cytotoxic function mediated among others by the action of perforins and granzymes. Additionally, the release of pro-inflammatory cytokines as TNF-α and IFN-γ could affect the local microenvironment, contributing to the exacerbation of the initial inflammatory process triggered by Stx2.

## Data Availability Statement

The raw data supporting the conclusions of this article will be made available by the authors, without undue reservation.

## Ethics Statement

The studies involving human participants were reviewed and approved by the Biosafety and Research Review Boards of the Instituto de Medicina Experimental–CONICET, Academia Nacional de Medicina and the Ethical Committee of the Institutos de la Academia Nacional de Medicina and the Universidad de Buenos Aires. Written informed consent to participate in this study was provided by the participants or participants’ legal guardian/next of kin.

## Author Contributions

Material preparation and experiments: DR, MR, CS and IK. HGEC culture: FG and RA. Critical revision of the manuscript and results discussion: CI and MA. Experimental design, data interpretation, and manuscript writing: CJ. All authors contributed to the article and approved the submitted version.

## Funding

This work was supported by grant from Agencia Nacional de Promoción Científica y Tecnológica (PICT2016/700 and PICT2019/255) and Consejo Nacional de Investigaciones Científicas y Técnicas.

## Conflict of Interest

The authors declare that the research was conducted in the absence of any commercial or financial relationships that could be construed as a potential conflict of interest.

## Publisher’s Note

All claims expressed in this article are solely those of the authors and do not necessarily represent those of their affiliated organizations, or those of the publisher, the editors and the reviewers. Any product that may be evaluated in this article, or claim that may be made by its manufacturer, is not guaranteed or endorsed by the publisher.

## Abbreviations

HGEC, human glomerular endothelial cells; HMBPP, (E)-4-hydroxy-3-methyl-but-2-enyl pyrophosphate; HUS, hemolytic uremic syndrome; IPP, isopentenyl pyrophosphate; Stx, Shiga toxin; STEC, Stx-producing *Escherichia coli*.
