# Supplementary material for: Human Glomerular Endothelial Cells Treated With Shiga Toxin Type 2 Activate γδ T Lymphocytes
Source: Front Cell Infect Microbiol. 2021 Nov 25;11:765941. doi: 10.3389/fcimb.2021.765941 (PMC8656354; doi:10.3389/fcimb.2021.765941)
Supplement: Supplementary file 1 [file Presentation_1.pdf]

Supplementary material

γδ T cells and HGEC co-culture assay

Purified γδ T cells were resuspended in M199 medium supplemented with 10% FBS (growth-arrested conditions). Then, $0.6x10^5$ γδ T cells were co-cultured with HGEC monolayers previously stimulated or not with Stx2a (0.01 ng/ml, 24h). The experiments were done in growth-arrested conditions medium without Stx2a. As a control, γδ T cells were cultured alone. After overnight incubation at 37°C, supernatants were recovered for ELISA analysis, and cells were treated with 5 mM EDTA to dissociate γδ T lymphocytes from HGEC for their subsequent immunostaining and flow cytometry analysis.

Treatment of γδ T cells with HGEC conditioned medium

Conditioned medium was obtained from confluent monolayers of HGEC pre-treated or not with Stx2a (0.01 ng/ml, 24 h), and storage until use. $0.6x10^5$ purified γδ T cells were resuspended in growth-arrested conditions medium and seeded in 96 round bottom well plates. Afterward conditioned medium was added, at a final concentration of $1x10^6$/ml. As control γδ T cells were incubated with growth-arrested conditions medium alone (basal). After overnight incubation at 37°C, γδ T cells were recovered for immunostaining and supernatants for ELISA analysis.

Cell-surface CD69 expression

Recovered γδ T cells ($0.6x10^5$) from previously described assays were incubated with saturating concentrations of mouse monoclonal antibodies anti-human CD69 conjugated to PE.Cy5 in PBS containing 0.5% bovine serum albumin (BSA) and 2 mM EDTA (staining buffer) (30 minutes at 4°C). Then, cells were washed with 300 µl of staining buffer, then washed with PBS, and fixed in 1% paraformaldehyde. Afterward samples were analyzed by flow cytometry (FACSCalibur, BD Bioscience, CA, San Diego).

Cytokine production assay

IFN-γ and TNF-α production by γδ T cells ($0.6x10^5$), were quantified by ELISA in the supernatants recovered from the previously described assays, following conventional protocols provided by the manufacturer (**Supplementary material, Table 2**).

For IL-6, IL-8 and TNF-α production by HGEC, supernatants were collected after 24 hours of incubation with or without Stx2a (0.01 or 1 ng/ml) in growth-arrested conditions media, according to the provider recommendations (**Supplementary material, Table 2**).

CD107a expression

γδ T cells were cultured with conditioned media obtained from monolayers of HGEC pre-stimulated or not with Stx2a (0.01 ng/ml, 24 h) as mentioned before, in the presence of saturating concentrations of mouse monoclonal antibodies anti-human CD107a conjugated to PE and monensin (2 µM) for 5 h at 37°C as previously described (Alter G et al., 2004). After the incubation time, cells were recovered, washed with 300 µl of staining buffer, then washed with PBS, and fixed in 1% paraformaldehyde. Afterward samples were analyzed by flow cytometry.

Intracellular cell staining of perforin and flow cytometry

After 4 h of incubation with the conditioned medium obtained from monolayers of HGEC pre-stimulated or not with Stx2a (0.01 ng/ml, 24 h), γδ T cells ($0.6x10^5$) were recovered and fixed in 2% paraformaldehyde for 20 minutes at 4°C. Then, cells were washed with PBS and permeabilized with PBS containing 0.5% BSA and 0.05% saponin

(permeabilization buffer), for 20 minutes at 4°C. After incubation, cells were centrifuge and immunostained with saturating concentrations of mouse monoclonal antibodies anti-human perforin in permeabilization buffer, for 30 minutes at 4°C. Then, cells were washed with 300 µl of permeabilization buffer, and after that washed in PBS, fixed with 1% paraformaldehyde, and analyzed by flow cytometry.

For the intracellular staining of TNF-α, γδ T cells ($0.6x10^5$) cultured with monolayers of HGEC (final concentration: $1x10^6$/ml), were treated with Brefeldin A (1µg/ml). After 5 hours of incubation, cells were recovered with PBS/5 mM EDTA, centrifuge, fixed in 1% paraformaldehyde (15 minutes at 4°C), and permeabilized with permeabilization buffer (20 minutes at 4°C). Afterward, cells were centrifuged, incubated with saturating concentrations of mouse monoclonal antibodies anti-human TNF-α conjugated to PE, in permeabilization buffer (30 minutes at 4°C). Then, cells were washed with 300 µl of permeabilization buffer, and then washed in PBS, fixed with 1% paraformaldehyde, and analyzed by flow cytometry. A gate based on size was done in the analysis to evaluate the expression of TNF-α in the γδ T cells.

Intracellular perforin expression by confocal microscopy

HGEC were seeded on fibronectin-coated glass coverslips (12 mm), overnight at 37°C. The next day, cells were stimulated or not with Stx2a (0.01 ng/ml) for 24 h. After that, the coverslips were washed with PBS, and γδ T cells ($0.6x10^5$) were incorporated and incubated at 37°C in 5% CO_2 for 5 h. After incubation, the coverslips were carefully washed with PBS to discard non-adherent cells, and then adherent cells were fixed in 2% paraformaldehyde and stained for perforin. Briefly, after fixation, the samples were permeabilized with permeabilization buffer for 20 minutes. Afterward, samples were incubated with saturating concentrations of mouse monoclonal anti-human perforin or the corresponding isotype control, in permeabilization buffer for 45 minutes at 4°C. After that, cells were washed with permeabilization buffer and incubated with a DyLight549-goat anti-mouse IgG antibody (9 µg/ml) for 30 minutes at 4°C. Finally, samples were washed with permeabilization buffer and fixed in 1% paraformaldehyde, and the coverslips were mounted onto glass slides using Fluoromount-G solution. Immunofluorescence images were acquired with a FluoView FV1000 confocal microscope (Olympus, Tokyo, Japan) using a Plapon 60X/1.42 NA oil immersion objective. Images were analyzed using FIJI software (National Institutes of Health, Bethesda, MD).

Neutral red cytotoxicity assay: HGEC monolayers were treated or not during 24 h with Stx2a (0.01 and 1 ng/ml), in growth-arrested conditions. After treatment, freshly diluted neutral red (10 µg/ml) was added to the cells and incubated for an additional 1 h at 37°C in 5% CO_2. Cells were then washed and fixed with 1% $CaCl_2$ + 1% formaldehyde, and lysed with 1% acetic acid in 50% ethanol to solubilize the neutral red. Absorbance was measured in an automated plate spectrophotometer at 540 nm. Results were expressed as a percentage of cell viability, where 100% represents cells incubated under identical conditions but without toxin.

Statistical Analysis

Statistical analysis was performed using GraphPad Prism v6.00 for Windows, GraphPad Software (La Jolla, CA, USA). Statistical significance was defined as $p<0.05$, by using nonparametric tests, with Dunn posttest for multiple competitions when necessary.

Supplementary material, figure 1

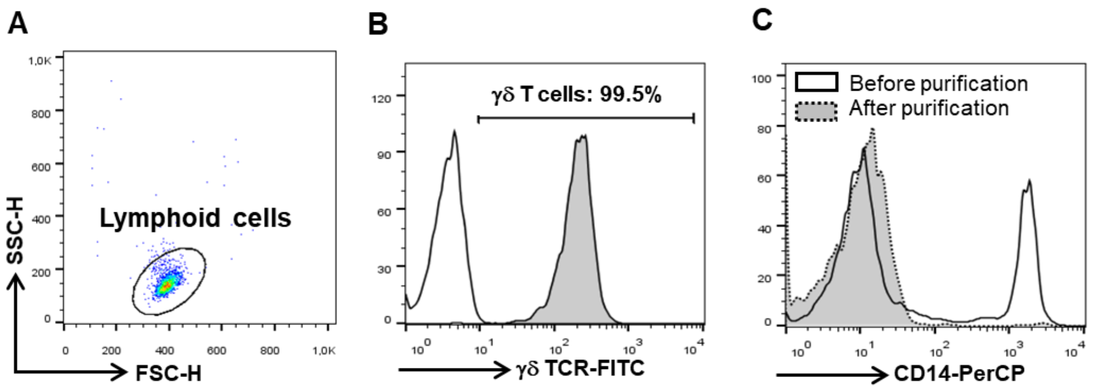

Supplementary figure 1: Purity of γδ T cells isolated from peripheral blood. (**A**) Representative dot plot (SSC-H vs FSC-H) of cells obtained after purification by employing the anti-TCR γδ MicroBead isolation kit. (**B**) Level of γδ TCR-FITC+ cells, analyzed in the T cell gate show in panel (A). (**C**) CD14 expression in peripheral blood mononuclear cells obtained after Ficoll-Hypaque gradient centrifugation (white histogram), compared with isolated γδ T cells (gray histogram).

Supplementary material, figure 2

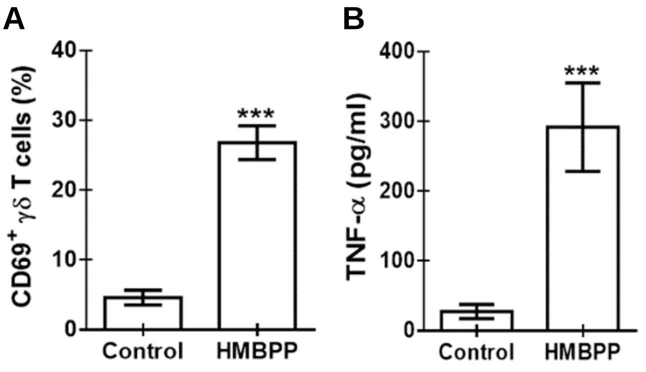

Supplementary figure 2: Purification procedure of γδ T cells did not affect their capability to be activated. Purified γδ T cells were stimulated with the specific agonist HMBPP (1 µM). After o.n. incubation, the expression of CD69 was analyzed by flow cytometry (**A**) and the production of TNF-α (**B**) by ELISA en cell supernatants. Results are shown as the mean ± SEM. n=14, biological replicates. ***$p<0.001$, Wilcoxon test.

Supplementary material, figure 3

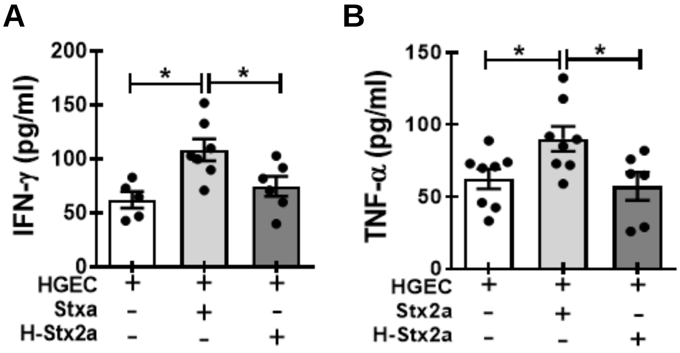

Supplementary figure 3: Heat-inactivated Stx2a did not stimulates γδ T cells. Purified γδ T cells were stimulated with conditioned medium obtained from HGEC treated or not with Stx2a (0.01 ng/ml, 24 h), heated (H-Stx2a) or not at 95°C for 3 h. After o.n. incubation of γδ T cells with those media, the production of IFN-γ (n=6) (**A**) and TNF-α (n=8) (**B**) was analyzed by ELISA en cell supernatants. Results are shown as the mean ± SEM, biological replicates. *$p<0.05$, Kruskal-Wallis test, with Dunn´s posttest.

Supplementary material, Table 1

A) Blood donors to purified γδ T cells:

**Number**	62 samples
**Average age**	38 years old
**Inclusion criteria**	1) The donor of blood must be between 18 and 65 years old. Minors between 16 and 18 years old must have the written and signed authorization of their parents or legal representatives, expressing their consent to the donation process.<br>2) Hg ≥ 12.5 g/dl<br>3) Hematocrit ≥ 38%.<br>4) Beats per minute between 50 and 100.<br>5) Systolic blood pressure, between 90 and 180 mmHg. Diastolic pressure between 60 and 100 mmHg. People who have no other health considerations and who are taking medications to control their blood pressure can donate blood if their blood pressure is within acceptable limits.<br>6) Body weight equal to or greater than 50 kg.
**Exclusion criteria**	1) Having had viral hepatitis after age 10, other than Hepatitis A.<br>2) Have or have had clinical or laboratory evidence of infections by *Tripanosoma cruzi*, HIV, HTLV, HCV, and/or HBV.<br>3) Injecting drug users not prescribed by doctors.<br>4) Persons who suffer from Hemophilia or are hemodialysis or periodically receive transfusions of blood, its components or derivatives.<br>5) Have had repeatedly suffered from syphilis or gonorrhea. Those potential donors who report having suffered a single episode with complete and adequate treatment may be included in a readmission protocol with a medical interview and a negative screening test.<br>6) Are at risk for Creutzfeldt-Jakob disease, or its variant. Have a family history of the disease.<br>7) Have received pituitary hormone of human origin between 1958 and 1966.<br>8) Has received a brain tissue or membrane transplant.<br>9) Have been reside for more than one year (adding all the periods of stay) in the United Kingdom during the period from 1980 to 1996, or in countries that have had foci of infection by CJ V.<br>10) Not having suffered, or have been at risk of contracting infections liable to be transmitted by transfusion (ITT). Information related to travel or stay in areas with a high prevalence of endemic ITTs (leishmania, borrelia, dengue, Variant of the agent of Creutzfeldt-Jakob disease, West Nile virus, among others) should be collected.<br>11) Pregnancy contraindicates donation. Women will be excluded for 6 weeks after a normal delivery, 12 months after a caesarean section or an abortion followed by an evacuation curettage. It is recommended that nursing mothers do not donate blood.<br>12) People who have undergone endoscopies will be excluded for a period of 6 months. Regarding laparoscopies and surgeries, a medical evaluation is necessary before accepting as a donor. When it comes to uncomplicated surgeries, it should be postponed for six months after the intervention. The deferral should be extended to 12 months if the person received transfusions.<br>14) At the time of evaluation, no show signs or symptoms of fever.

	13) They are disqualified for 12 months (temporarily) as donors of blood or blood components, those who have received tattoos, non-sterile skin piercing and/or acupuncture or suffered occupational accidents with exposure to blood or secretions (punctures or contact with them through mucous membranes or wounds).<br>14) Potential donors who have received blood transfusions, components or blood derivatives must be deferred for twelve months.

B) Kidney fragment donors for HGEC isolation:

**Number**	4 samples
**Average age**	5 years old
**Clinical status**	Nephrectomie due to segmental uropathies or tumors in one pole of kidney
**Inclusion criteria**	Normal creatine
**Exclusion criteria**	Necrotic or fibrous tissue were not processed

Supplementary material, Table 2

Reagents and antibodies

Reagent	Supplier	Catalog number
anti-human CD107a-PE	BioLegend (San Diego, California, USA)	328607
anti-human CD14-PE	BioLegend (San Diego, California, USA)	325606
anti-human CD69-PE.Cy5	BD Bioscience (San Jose, CA, USA)	555532
anti-human perfotin-PerCP-Cy5.5	BioLegend (San Diego, California, USA)	308114
anti-human TNF-α-PE	BioLegend (San Diego, California, USA)	502909
anti-TCRγδ MicroBead kit	Miltenyi Biotec (Germany)	130-050-701
DyLight™ 549 AffiniPure Goat Anti-Mouse IgG	Jackson ImmunoResearch Inc. (Chester, PA, USA)	115-505-209
endothelial cell growth supplement	Sigma (St. Louis, MO, USA)	E2759
Etanercept	Wyeth Pharmaceuticals (Hampshire, England)	----
fetal bovine serum	Invitrogen (Carlsbad, CA, USA)	16000-044
Ficoll-Hypaque	GE Healthcare Bio-Sciences AB (Uppsala, Sweden)	17-1440-03
HMBPP	Sigma (St. Louis, MO, USA)	95098
BD OptEIA™ Human IFN-γ ELISA Set	BD Bioscience (San Jose, CA, USA)	555142
BD OptEIA™ Human IL-6 ELISA Set	BD Bioscience (San Jose, CA, USA)	555220
BD OptEIA™ Human IL-8 ELISA Set	BD Bioscience (San Jose, CA, USA)	555244
BD OptEIA™ Human TNF-α ELISA Set	BD Bioscience (San Jose, CA, USA)	555212
L-glutamine	GIBCO (Waltham, MA, USA)	25030081
M199	Sigma (St. Louis, MO, USA)	M5017
neutral red	Sigma (St. Louis, MO, USA)	N7005
RPMI 1640	Invitrogen (Carlsbad, CA, USA)	22400-089
Stx2a	The Phoenix Lab. Tufts Medical Center (Boston, MA)	----
